# A Plant's Guide to Surviving the Chicxulub Impact

**DOI:** 10.1371/journal.pbio.1001948

**Published:** 2014-09-16

**Authors:** Jonathan M. Chase

**Affiliations:** Freelance Science Writer, Saint Louis, Missouri, United States of America

What comes to your mind when you hear the word “extinction?” Perhaps you imagine a critically endangered Giant Panda gnawing on bamboo or a tiger crouching in the brush amidst the seemly endless degradation of their habitats. Or perhaps you imagine herds of dinosaurs and other creatures that we only know from fossils that are millions of years old. Odds are pretty good that an image of a plant wasn't the first (or the second, third, or fourth) thing to pop into your head. And yet, there are about as many plant species as animals species that are considered to be critically endangered by extinction (and there would probably be many more if the data were available). And what about the Chicxulub impact, the massive asteroid that collided with the Earth about 66 million years ago and almost certainly instigated the mass extinction that drove the non-avian dinosaurs and about 75% of the species on the planet extinct? Well, it turns out that this impact, at the boundary between the Cretaceous (K) and Paleogene (Pg) periods, has been implicated in the extinction of approximately half of all plant species that existed in North America at the time.

There are two roads to extinction. First, a species can go extinct simply because of bad “luck.” Perhaps a species experiences a few years of poor reproduction and is then subject to a freak storm that causes high mortality of the few individuals left. As an analogy, think of gambling at the slot machines, which involves no skill or predictability in winning or losing. You walk to a machine with a handful of coins and, more often than not, you walk away a few minutes later empty-handed. The second road to extinction happens when a species' traits are poorly matched to its environment and that species' ability to replace itself through reproduction is outpaced by its mortality rate. A sudden shift in climate, for example, can create a hostile environment for species that were once well suited to a particular location, and they must either migrate towards more favorable conditions or face extinction. To go back to the gambling analogy, a poker player whose skills are poorly matched to others at the table will rapidly go broke despite the random likelihood of being dealt a good hand on occasion.

**Figure 1:**
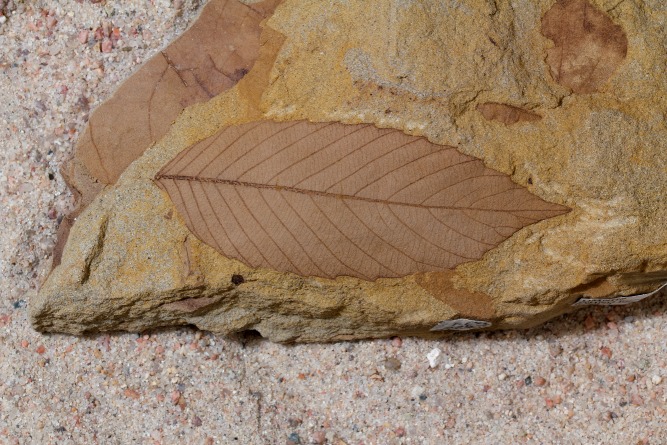
Fossil leaves record information about plant ecological strategies. Seen here is a Late Cretaceous specimen from the Hell Creek Formation, morphotype HC62, taxon *“Rhamnus” cleburni*. Specimens are housed at the Denver Museum of Nature and Science in Denver, Colorado. *Image credit: Benjamin Blonder*.

So, what happened after the Chicxulub impact on what is now the Yucatán Peninsula 66 million years ago? Were the few winners that survived the mass extinction event just lucky? Or did they have traits that somehow made them better suited to survive the decades-long impact winter that followed? And if the latter, which traits were favored or disfavored? Ecologists studying contemporary extinctions have been asking these sorts of questions for decades and have devised a number of statistical tools to disentangle the two roads towards extinction. However, similar approaches have not been so forthcoming in paleoecology, in which data are often incomplete. In this issue of *PLOS Biology*, Blonder and colleagues overcome this barrier by combining modern ecological approaches with data from a diverse and exceptionally well-preserved set of fossilized plant leaves from North Dakota (United States) that spanned a 2.2 million-year period bracketing the K–Pg mass extinction event (Figure 1).

The primary goal of Blonder and colleagues' work was to determine whether plant species that went extinct during the K–Pg mass extinction event were non-random with respect to their traits relative to those that persisted. Taking their cue from studies on extant plant species and their distributions, Blonder and colleagues measured two key plant leaf traits—leaf mass per area (LMA, indicating the amount of carbon invested per leaf area) and leaf minor vein density (VD, indicating the ability to move carbon and water in and out of leaves)—on hundreds of fossilized plant leaves. These two traits provide insights into where a given plant species is along the “leaf economics spectrum.” On the one hand, leaves can be “fast return,” indicated by relatively low LMA and high VD; these leaves are relatively cheap to make and can allow plants to take up resources rapidly when conditions are favorable (e.g., warmer and wetter) but are lost (along with their energetic maintenance costs) when conditions are less favorable. This strategy is exemplified by many deciduous species that acquire resources in variable environments. On the other hand, they can be “slow return,” indicated by higher LMA and lower VD; these are more costly to make and are longer lived. Species on this end of the spectrum tend to live in less variable environments and are often evergreen.

Blonder and colleagues estimated the VD and LMA of leaf fossil assemblages starting around 1.4 million years before the K–Pg boundary and going up to 0.8 million years after it. They found a more than 10% decrease in the average LMA and a nearly 70% decrease in its variance across the K–Pg boundary, mostly because species with particularly high LMA that were abundant in the Cretaceous largely disappeared in the Paleogene. On the other hand, they found a nearly 30% increase in the VD of the plant assemblage across the boundary (but no change in variance), and this occurred primarily because of a loss of species with low VD that lived during the Cretaceous but went extinct during the K–Pg event. Both of these results are consistent with a hypothesis that the lower light levels and high climatic variability that resulted from the impact winter after the bolide impact resulted in a directional shift towards species with fast strategies (low LMA, high VD) and away from species with slower strategies (i.e., angiosperms that are evergreen, such as holly and rhododendrons).

Importantly, Blonder and colleagues were cognizant of a number of potential sampling biases that might have influenced their results and found that most of their results were robust to these biases. The one bias that might have influenced their results, however, was the preservation sites themselves. Cretaceous sites tended to come from floodplains, whereas Paleogene sites were more likely to come from channels of flowing water. When they corrected for this potential bias by comparing traits from within each type of site, the VD results remained significant, but the LMA results were no longer so because of low sample sizes. In the end, the results showed that changes in VD were quite strong and those in LMA moderate, showing a clear shift in plant functional traits indicating that the K–Pg mass extinction was strongly selective, rather than random, in determining its victims.

The results from Blonder and colleagues indicating that species on the slow-return side of the leaf economics spectrum disproportionately succumbed to extinction also suggest some tantalizing, albeit speculative, insights into how the ecosystems in which these species were embedded may have changed as a result of the mass extinction event. The shift towards plant communities with a higher proportion of fast-return species could have led to higher rates of ecosystem functioning (e.g., productivity, decomposition rates, and water cycling), and this could have fed back to influence a number of local and global processes. Regardless, by extending concepts and tools from modern ecological study into the deep past, the study by Blonder and colleagues provides a deeper understanding of what determines the winners and losers during a mass extinction and may even provide insights into the ongoing mass extinction that appears to be resulting from the dramatic and multifaceted global changes caused by humans.

**Blonder B, Royer DL, Johnson KR, Miller I, Enquist BJ, et al. (2014) Plant Ecological Strategies Shift Across the Cretaceous–Paleogene Boundary.** doi:10.1371/journal.pbio.1001949

